# Deciphering Key Foreign Body Reaction-Related Transcription Factors and Genes Through Transcriptome Analysis

**DOI:** 10.3389/fmolb.2022.843391

**Published:** 2022-03-08

**Authors:** Wei Liu, Shaoheng Xiong, Jing Du, Yajuan Song, Tong Wang, Yu Zhang, Chen Dong, ZhaoSong Huang, Qiang He, Zhou Yu, Xianjie Ma

**Affiliations:** Department of Plastic Surgery, Xijing Hospital, Fourth Military Medical University, Xi’an, China

**Keywords:** foreign body reaction, fos, mmp9, hub genes, silicone implants, fibrous capsule, transcription factors, transcriptome

## Abstract

**Background:** Silicone implants are widely used in the field of plastic surgery for wound repair and cosmetic augmentation. However, molecular mechanisms and signaling pathways underlying the foreign body reaction (FBR) of a host tissue to the silicone require further elucidation. The purpose of this study was to identify key FBR-related transcription factors (TFs) and genes through transcriptome analysis.

**Methods:** We used a rat model with a subcutaneous silicone implant in the scalp and performed high throughput sequencing to determine the transcriptional profiles involved in the FBR. The function was analyzed by Gene Ontology (GO) and the Kyoto Encyclopedia of Genes and Genomes (KEGG) pathway-enrichment analysis. A protein-protein interaction (PPI) network of differentially expressed mRNAs (DEmRNAs) was constructed to identify the hub genes and key modules and to determine the regulatory TF-mRNA relationships. In addition, the hub gene and transcript expression levels were determined by Quantitative Reverse Transcription polymerase Chain Reaction (qRT-PCR). Myofibroblasts differentiation and macrophage recruitment were identified by immunofluorescence. The protein expression of MMP9 was detected by immunohistochemistry and Western blot.

**Results:** We identified ten hub genes (*Fos, Spp1, Fn1, Ctgf, Tlr2, Itgb2, Itgax, Ccl2, Mmp9*, and *Serpine1*) and 3 TFs (FOS, IRF4, and SPI1) that may be crucial (particularly FOS) for the FBR. Furthermore, we identified multiple differentially expressed genes involved in several important biological processes, including leukocyte migration, cytokine‒ cytokine receptor interaction, phagocytosis, extracellular matrix (ECM) organization, and angiogenesis. We also identified potentially significant signaling pathways, including cytokine‒cytokine receptor interaction, phagosome, ECM‒receptor interaction, complement and coagulation cascades, the IL-17 signaling pathway, and the PI3K‒Akt signaling pathway. In addition, qRT-PCR confirmed the expression patterns of the TFs and hub genes, Western blot and immunohistochemistry validated the expression patterns of MMP9.

**Conclusion:** We generated a comprehensive overview of the gene networks underlying the FBR evoked by silicone implants. Moreover, we identified specific molecular and signaling pathways that may perform key functions in the silicone implant-induced FBR. Our results provide significant insights into the molecular mechanisms underlying silicone-induced FBR and determine novel therapeutic targets to reduce complications related to silicone implantation.

## 1 Introduction

Silicone implants have been used for cosmetic and reconstructive purposes since their introduction in 1962. In fact, they are the most popular and ideal material for augmentation procedures (Albornoz et al., 2013), such as breast implants, tissue expanders, and nasal prostheses. Owing to the wide-spread application and large-scale population exposure, multiple studies have investigated their safety, primarily focusing on complications of implant‒host interactions (Janowsky et al., 2000; Caplin et al., 2021).

Silicone implants are recognized as foreign objects by the host immune system, thereby initiating a series of interactions at the implant‒host interface and inducing a foreign body reaction (FBR). It is a primary reaction of the innate immune system and is evoked upon implantation of foreign materials in the host body (Anderson et al., 2008). The silicone implant-related FBR may lead to common silicone-induced complications (capsule formation and contracture) and increase the risk of developing connective tissue diseases or even autoimmune diseases (Goren et al., 2015; Cuomo, 2021). Therefore, understanding the pathophysiological mechanisms underlying the FBR may help reduce the complications associated with silicone implantation.

Much effort has been put into researching the interactions between silicone implants and host tissues. Previous studies have elucidated many aspects of the five FBR phases: protein adsorption, acute inflammation, chronic inflammation, foreign body giant cell formation, and fibrous capsule formation (Luttikhuizen et al., 2006; Kastellorizios et al., 2015). Moreover, FBR is influenced by numerous factors, such as immune components of the implant materials, cell behavior in the localized immune microenvironment, and cytokine and inflammatory protein productions induced by implants (Veiseh and Vegas, 2019). However, the molecular mechanisms and signaling pathways involved in the FBR of host tissues to silicone have not been elucidated.

In this study, we used a comprehensive biological pipeline to explore key molecular signatures of FBR pathophysiology induced by silicone implants. We used a high-throughput sequencing method to determine the transcriptome profiles associated with the FBR of the host skin in response to a subcutaneous silicone implant in a rat model. Bioinformatics analyses were performed to validate key differentially expressed mRNAs (DEmRNAs). Furthermore, cluster and pathway analyses were conducted to investigate the possible molecular mechanisms and signaling pathways involved in a silicone implant-induced FBR. Subsequently, we generated a protein-protein interaction (PPI) network to predict key modules of the FBR. Hub genes were identified, and transcription factor (TF)‒mRNA regulatory relationships were deciphered. In addition, qRT-PCR was performed to verify the expression of the hub genes and TFs, immunohistochemistry and Western blot were used to detect the expression patterns of MMP9.

## Materials and Methods

### Animal Experiments

Six-week-old male Sprague–Dawley rats were purchased from the Experimental Animal Center of the Fourth Military Medical University and were fed under specific pathogen-free conditions. A round silicone sheet of 1.0 cm diameter was customized by Wanhe Plastic Materials Co., Ltd. (Guangzhou, China). Eight rats were randomly divided into two groups: a silicone-implanted group (*n* = 4) and a control group (*n* = 4). The silicone sheet was surgically implanted under the scalp of rats that belonged to the silicone-implanted group, whereas the control group rats did not receive any implants. The experimental protocol was approved by the animal experiment ethics committee of the Fourth Military Medical University (Xi’an, China) (permit no. IACUC-20120117), and animal experiments were performed according to the guidelines of the Animal Care Committee. Further details are provided in the Supplementary Materials and Methods.

### Tissue Collection

At 30-days post-surgery, all the rats were anesthetized and the fibrous capsule-containing scalp was dissected out. One part of the scalp tissue sample was fixed in 10% neutral buffered formalin and was subsequently embedded in paraffin. The remaining tissues were stored in RNAlater (Invitrogen, Waltham, MA, United States) at −20°C.

### Hematoxylin and Eosin (H&E) Staining and Immunofluorescence

The tissue sections were stained with H&E according to routine procedures and were also subjected to immunohistochemistry. First, they were subjected to an antigen retrieval step at 96°C for 20 min in a citrate buffer at pH 6.0. Subsequently, they were blocked with phosphate buffer saline (PBS) with 5% bovine serum albumin for 1 h and incubated overnight with primary antibody at 4°C in a humid chamber. The antibodies were mouse anti-rat CD68 (1:100, ab31630, Abcam, Cambridge, United Kingdom), rabbit anti-rat alpha-smooth muscle actin (1:100, 14395-1-AP, ProteinTech Group, Wuhan, China), rabbit anti-rat iNOS (1:25, ab31630, Abcam, Cambridge, United Kingdom), rabbit anti-rat CD206 (1:200, ab64693, Abcam, Cambridge, United Kingdom). The sections were then incubated with fluorescently labeled secondary antibodies (antibody to mouse or rabbit) for 1 h at 37°C in a humid chamber (1:1,000, Invitrogen, Carlsbad, CA, United States). Cell nuclei were counterstained with 10 μg/ml of DAPI (Solarbio, Beijing, China). Fluorescence was detected using a Nikon C2 Confocal microscope (Nikon, Tokyo, Japan) with a ×20 and ×40 objective.

### Library Construction, Examination, Clustering and Sequencing

A total of 2 μg RNA per sample was used to generate cDNA libraries using the NEBNext ^®^ Ultra™ RNA Library Prep Kit for Illumina ^®^ (#E7530L, New England BioLabs Inc, Ipswich, MA, United States), as per the manufacturer’s recommendations. The RNA concentration of the library was measured for preliminary quantification and then diluted to 1 ng/ul. Subsequently, insert size was quantified accurately. Clustering of the index-coded samples was performed on a cBot cluster generation system using HiSeq PE Cluster Kit v4-cBot-HS (Illumina, San Diego, CA, United States), according to the manufacturer’s instructions. One silicone-implanted sample and one control sample were not considered for this step because of a clear separation between them and the other samples in their respective groups. After cluster generation, the libraries were sequenced using Illumina HiSeq 4,000 (Annoroad Co. Ltd.) and 150 bp paired-end reads were generated. Further details are described in the Supplementary Materials and Methods.

### Data Filtering and Alignment

The generated raw reads were processed by removing the adapter sequences and low-quality bases and N-bases using the Perl scripts (https://github.com/mdshw5/fastqp). Thereafter, the remaining high-quality clean reads were aligned to the reference genomes and annotation file (Ensembl, v. Rnor 6.0.87) using HISAT2 v2.1.0. Eventually, the read counts for each gene in each sample were counted by HTSeq v0.6.0. Furthermore, the fragments per kilobase million mapped reads were calculated to estimate the gene expression levels. Further details are described in the Supplementary Materials and Methods.

### Differential Gene Expression Analysis

Using DESeq2, we estimated the expression of each gene in each sample. The *p*-value was calculated and corrected. Gene transcripts with |log2FC| ≥ 1.5 and *p* < 0.05, q < 0.05 were classified as DEmRNAs. Volcano plots of DEmRNAs were prepared using the “ggplot2” library of the R software. The correlation coefficient of every two samples was calculated using the “pearson” function in the “stats” package, and the results were visualized in R using the “pheatmap” package. Further details are described in the Supplementary Materials and Methods.

### Principal Component Analysis (PCA)

We performed the PCA of our transcriptome data using the “prcomp” function of the “stats” package to assess the resemblance between samples. The obtained results were visualized in R using the “scatterplot” package. Further details are described in the Supplementary Materials and Methods.

### Gene Ontology (GO) and Kyoto Encyclopedia of Genes and Genomes (KEGG) analysis

Metascape (http://metascape.org) (Zhou et al., 2019) was used to conduct a GO and KEGG pathway enrichment analysis of DEmRNAs; the criteria for this analysis were a *p*-value < 0.01, a minimum count of 3, and an enrichment factor >1.5. A functional enrichment analysis was also performed using the GO (version:1.2)/KEGG (version:99.0) tools in Hiplot (https://hiplot.com.cn/advance/clusterprofiler-go-kegg). The most statistically significant term within a cluster was considered to represent that cluster. Further details are described in the Supplementary Materials and Methods.

### Gene Set Enrichment Analysis (GSEA)

We performed the GSEA (https://software.broadinstitute.org/gsea/index.jsp) using the GSEA software version 2.2.2.0. The H, C2, and C5 collections were used from the Molecular Signatures database (MSigDB v5.0). Moreover, a threshold of *p* < 0.05 was applied for analysis. Further details are described in the Supplementary Materials and Methods.

### PPI Network Analysis

The Search Tool for the Retrieval of Interaction Genes/Proteins (STRING v11.0, https://string-db.org/) database was used to construct a PPI network of DEmRNAs. We used Cytoscape 3.8.0 (https://cytoscape.org/) to visualize this network. Subsequently, the Molecular Complex Detection (MCODE) tool (https://apps.cytoscape.org/apps/mcode) was used to search for high modularity clusters within the network. Furthermore, the ClueGO plugin (https://apps.cytoscape.org/apps/cluego) was used to identify terms associated with the DEmRNAs of each cluster. Eventually, the hub genes were identified by the CytoHubba plugin (https://apps.cytoscape.org/apps/cytohubba). Further details are described in the Supplementary Materials and Methods.

### Transcription Factor Identification and TF–mRNA Regulation Relationship Construction

We identified interactive TFs using the Animal Transcription Factors database (Hu et al., 2019). Thereafter, ChIP Enrichment Analysis (ChEA): Transcription Factor Binding Site Profiles, JASPAR Predicted Transcription Factor Targets, ENCODE Transcription Factor Targets, and TRANSFAC Curated Transcription Factor Targets were used to predict the target genes. Transcripts of the predicted target genes were compared with the DEmRNAs to identify the overlapping DEmRNAs. Lastly, the TF–mRNA interactions were visualized by Cytoscape.

### Quantitative Reverse Transcription Polymerase Chain Reaction (qRT-PCR) Analysis

Total RNA from the tissues was extracted with the TRIzol reagent (Invitrogen, Camarillo, CA, United States). Subsequently, 1,000 ng of the extracted RNA was reversely transcribed into cDNA using the PrimeScript RT reagent Kit with gDNA Remover (Takara, Shiga, Japan). We performed qRT-PCR using the TB Green Premix Ex Taq II (Takara, Shiga, Japan) on a BIO-RAD CFX Connect Real-Time System (Bio-Rad, Munich, Germany). The relative expression levels of target genes were normalized with that of GAPDH using the 2^−ΔΔCt^ method. The hub genes (*Fos, Spp1, Fn1, Ctgf, Tlr2, Itgb2, Itgax, Ccl2, Mmp9,* and *Serpine1*) and TFs (FOS, SPI1, IRF4, MYOG, CTGF, MEF2C, and SREBF1) were analyzed by qRT-PCR. The primer sequences are listed in Supplementary Table S1.

### Immunohistochemistry

Sections were deparaffinized in xylene and rehydrated in a descending alcohol series followed by distilled water. Then the antigens were retrieved in Tris-EDTA buffer at 96°C for 20 min. The slides were treated with 3% hydrogen peroxide to quench endogenous peroxide activities for 15 min and tissues were incubated with the primary antibody at 4°C overnight. The primary antibody used was anti-MMP9 (10375-2-AP, Proteintech, China, 1:200) and the tissue was incubated with secondary antibody using Dako REAL EnVision/HRP, Rabbit/Mouse (Agilent Technologies, CA, United States) IHC kits for 1 h at 37 °C. The signal was visualized using Dako REAL EnVision Detection System, peroxidase/DAB+, Rabbit/Mouse (Agilent Technologies, CA, United States) kit; nuclei were counterstained with hematoxylin.

### Western Blot

Total proteins were extracted with RIPA lysis buffer (CWbiotech, Shanghai, China) supplemented with protease inhibitors and phosphatase inhibitors (CWbiotech, Shanghai, China). The protein concentration was measured by a BCA assay (CWbiotech, Shanghai, China). The samples were separated by electrophoresis on 8% SDS-PAGE and then transferred to polyvinylidene fluoride membranes (Millipore, Bedford, MA, United States). The membranes were blocked with PBST with 5% w/v BSA for 1 h at room temperature and then incubated with primary antibodies anti-MMP9 (10375-2-AP, Proteintech, China, 1:1,500) overnight at 4°C. The membranes were washed thoroughly with PBST. The membranes were incubated with the secondary antibody (CW0103, CWbiotech, China, 1:3,000) for 1 h at room temperature and rinsed thoroughly with PBST. Immune reactivity was detected using SuperSignal West Pico PLUS kit (Thermofisher Scientific, Waltham, MA, United States) under a Tanon 4,600 chemiluminescent imaging system (Tanon, Shanghai, China). Relative protein levels were calculated after normalization to GAPDH, which was used as a loading control.

### Statistical Analyses

Statistically significant differences between the two groups were determined by two-tailed unpaired t-tests and the Mann–Whitney *U* test. The results from at least three experimental repeats are presented as mean ± standard deviation (SD) in column graphs. The *p*-values < 0.05 were statistically significant. The data were plotted using GraphPad Prism 7.0 (GraphPad Software, San Diego, CA, United States).

## Results

### Silicone Implantation-Induced FBR Leads to Fibrous Capsule Formation

**FIGURE 1:**
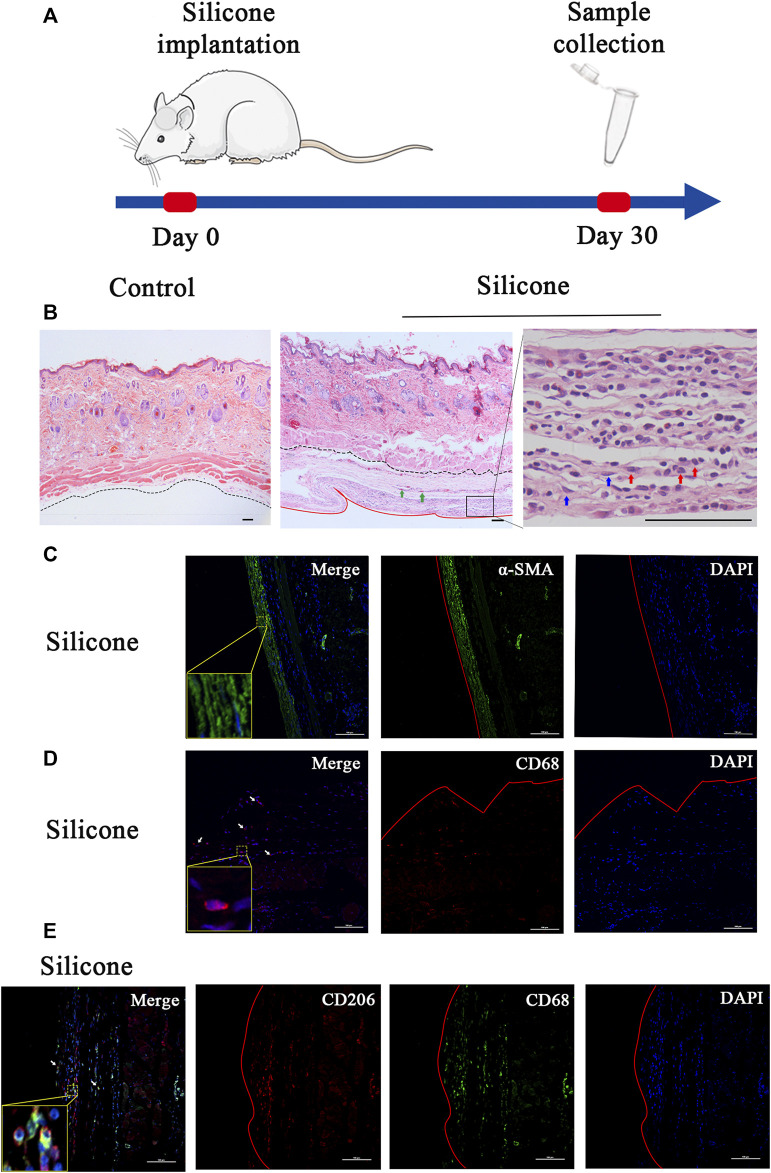
Overview of the study design and histological characteristics of fibrous capsule. **(A)** Schematic presentation of procedural timeline of silicone implantation. **(B)** H&E staining showed a fibrous capsule wrapped around the silicone implant. The fibrous capsule was located beneath the muscle layer. No capsule formed under the control skin. The black dotted line marks the boundary between the skin and fibrous capsule, the red solid line marks the interface between fibrous capsule and silicone implant. Green arrows point to blood vessels, blue arrows point to myofibroblasts, red arrows point to macrophages. **(C)** The immunofluorescence staining of α-SMA in capsule showed myofibroblast differentiation in silicone–implanted group. **(D)** The immunofluorescence staining of CD68 in capsule showed macrophage recruitment in silicone–implanted group. Arrows point to macrophages. **(E)** The immunofluorescence staining of CD68 and CD206 in capsule showed M2 macrophages. α-SMA: alpha-smooth muscle actin. Insets show high-magnification images. Scale bar = 100 μm. The red solid line marks the interface between fibrous capsule and silicone implant.

To understand the molecular changes related to the interactions between a host tissue and a silicone implant, we constructed a rat sub-scalp silicone-implanted model (Figure 1A). One month after the implantation, we evaluated the transcriptome profiles associated with the FBR in the silicone and control groups. General observations revealed a fibrous capsule that was wrapped around the silicone implant; this is a typical characteristic of FBR. Additionally, H&E staining of the silicone-implanted skin demonstrated fibrous capsule formation at the tissue‒implant interface. Numerous fibroblasts, myofibroblasts, macrophages, foreign body giant cells (FBGCs), and blood vessels were present in these capsules. However, the skin of the control rats exhibited none of these features (Figure 1B; Supplementary Figure S1).

Furthermore, we observed myofibroblast differentiation and macrophage recruitment in the skin tissue samples. In this regard, we performed immunofluorescence staining for α-SMA as a myofibroblast marker and for CD68 as a pan-macrophage marker. Consequently, we discovered that the cellular densities of α-SMA-positive cells and CD68-positive cells were higher in the silicone group than those in the control group (Figures 1C,D; Supplementary Figures S2A,B).

In addition, we tried to identify the phenotypes of macrophages during the FBR. Immunofluorescence staining with the traditional M1 marker (iNOS) or M2 marker (CD206) in combination with a pan-macrophage marker (CD68) was used to distinguish each macrophage phenotype in the fibrous capsule. The results, as shown in Figure 1E, demonstrated a substantial amount of CD68^+^/CD206^+^ double-positive M2 macrophages (Figure 1E; Supplementary Figure S2C) and a few CD68^+^/iNOS^+^ double-positive M1 macrophages (Supplementary Figure S2D).

### Silicone Implantation Modulates mRNA Expression Profiles in the Skin

**FIGURE 2:**
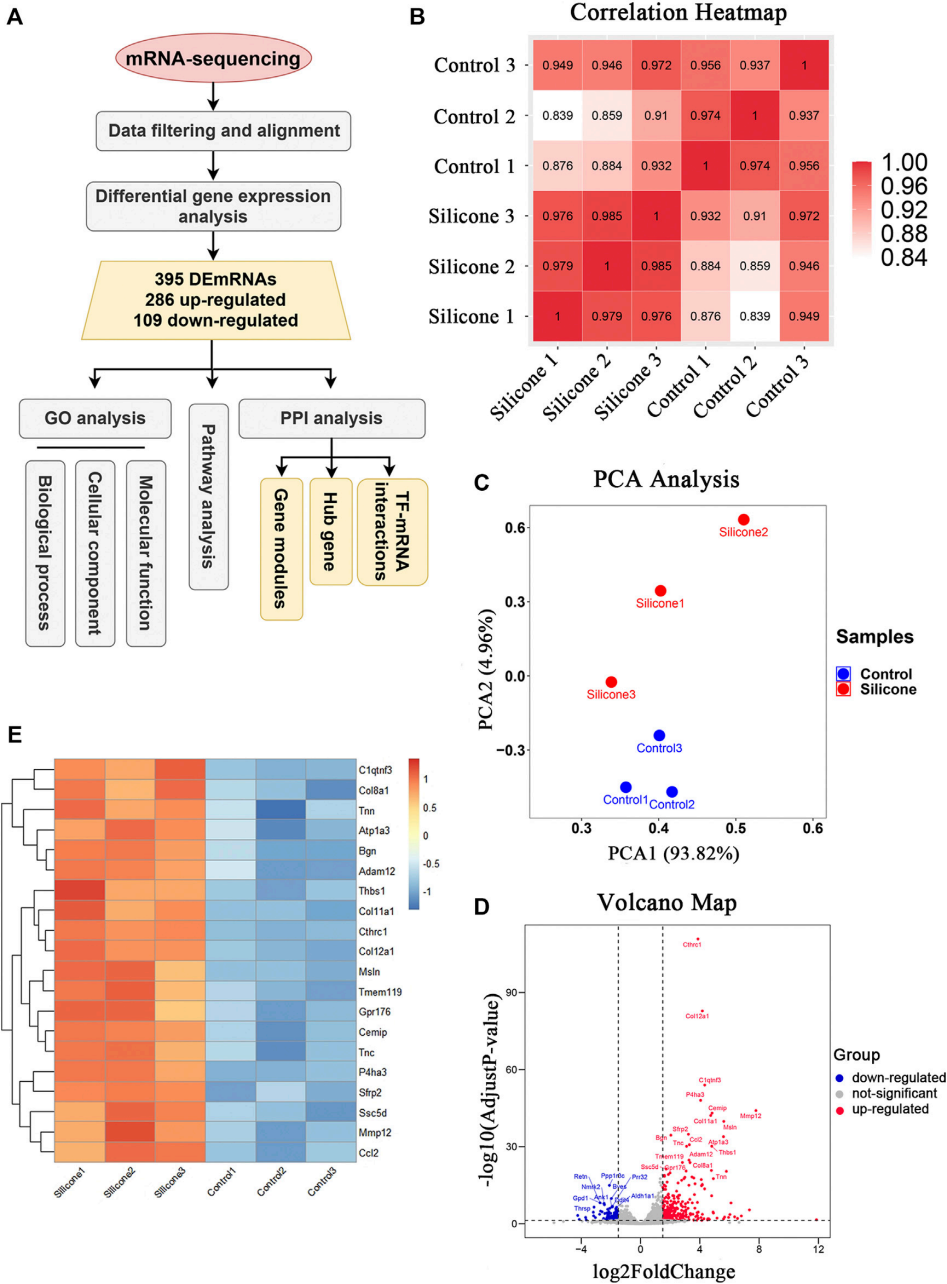
The quality control of samples and the identification of DEmRNAs between silicone group and control group. **(A)** Flowchart of data acquirement, processing, analysis, and validation. GO, Gene Ontology; DEmRNAs, differentially expressed mRNAs; PPI, protein-protein interaction; TF, transcription factors. **(B)** HeatMap of correlation analysis with unsupervised hierarchical clustering between silicone and control group. **(C)** Scatter plot of PCA analysis between silicone and control group. PCA, principal component analysis. **(D)** Volcano map of differentially expressed genes between silicone and control group. **(E)** HeatMap of top 20 DEmRNAs.

We investigated the potential molecular phenotype of the fibrous capsule related to FBR by dissecting the skin tissues surrounding the silicone implants from each group 1 month post-implantation. The RNA transcripts of these samples were bioinformatically analyzed (Figure 2A). We obtained 4.1 ± 1.9 million reads per sample after removing adaptor sequences and low-quality reads. The base accuracy rate of the Q30 score varied from 92.96 to 93.34% across the samples, indicating that the sample processing and sequencing was of high quality.

Unsupervised clustering revealed clear differences among the two groups. For instance, the skin samples from the silicone group clustered together based on the similarity of their gene expression profiles, which was different from that of the control samples. This confirmed the uniformity within each group and the independence between the groups (Figure 2B). Our PCA showed that silicone implantation had profound effects on gene expression. Thus, the RNA-seq data were credible (Figure 2C).

The comparative analysis identified 395 DEmRNAs between the silicone and control groups (|log2FC| ≥ 1.5 and *p* < 0.05, q < 0.05). Among them, 286 DEmRNAs were upregulated, while 109 DEmRNAs were downregulated (Supplementary Table S2). The volcano plot showed that these DEmRNAs were clearly distinct (Figure 2D). The top 20 up-regulated genes were displayed in Figure 2E. Collectively, these data indicated that silicone implantation in the rat scalp modulated the expression profiles of multiple genes in the skin, contributing to the FBR and fibrous capsule formation.

### Functional Enrichment and Pathway Analysis of DEmRNAs Caused by Silicone Implantation

**FIGURE 3:**
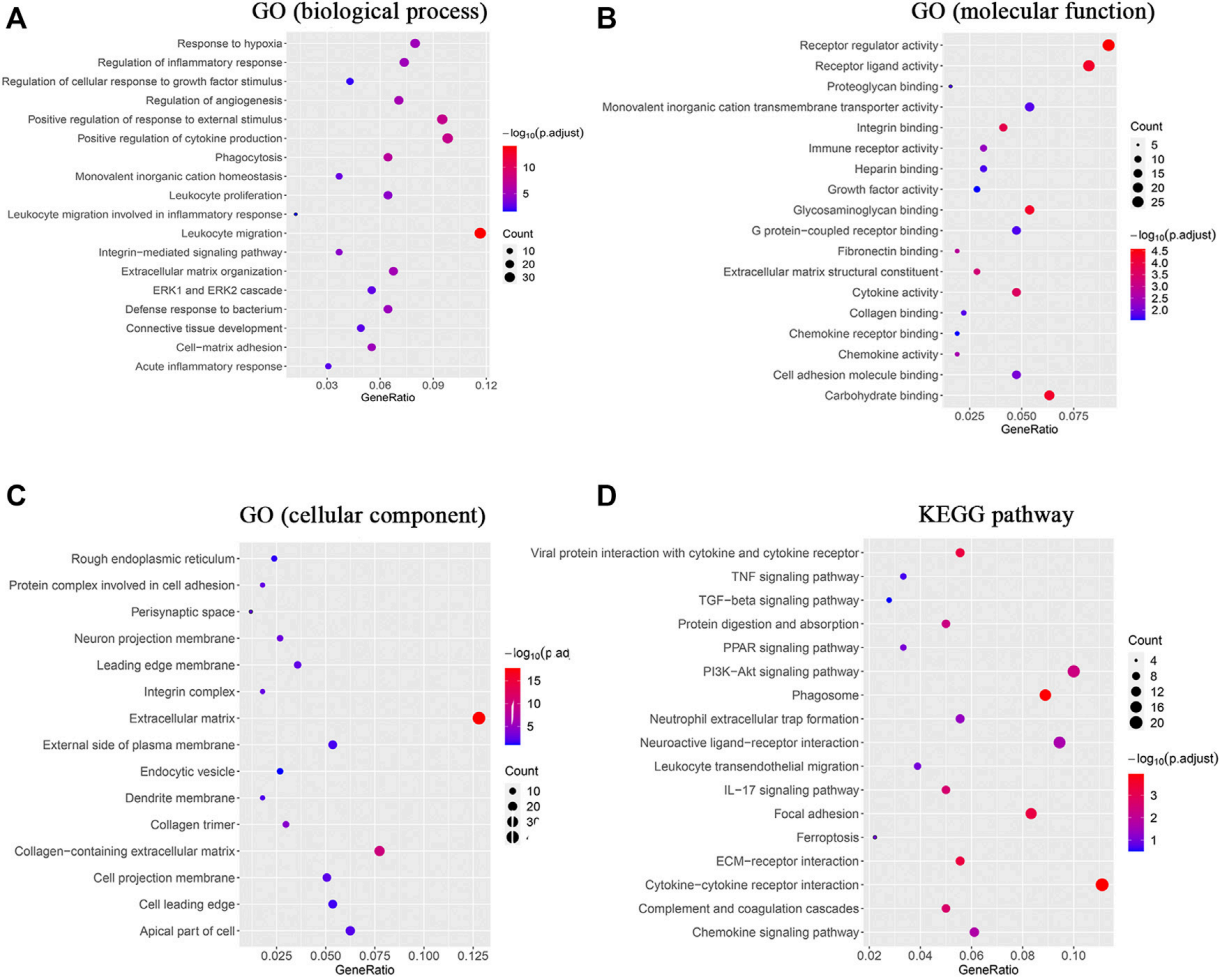
GO enrichment analysis and KEGG analysis revealed changes in the signaling pathways and molecular response of skin tissue during the FBR induced by silicone implants. **(A)** GO enrichment analysis of the DEmRNAs in biological processes. **(B)** GO enrichment analysis of the DEmRNAs in molecular functions. **(C)** GO enrichment analysis of the DEmRNAs in cellular components. **(D)** KEGG analysis of the DEmRNAs. Gene ratio indicates the number of DEmRNAs associated with the GO term divided by the total number of DEmRNAs. Dot size represents the number of DEmRNAs associated with the GO term and the color represents the negative value of log10 of adjusted *p*-value. KEGG, Kyoto Encyclopedia of Genes and Genomes.

To investigate the enrichment of functional terms of the identified DEmRNAs, GO functional enrichment analysis was performed. It revealed significant enrichment of the DEmRNAs in various biological processes (Figure 3A): leukocyte migration (GO:0050900), positive regulation of cytokine production (GO:0001819), positive regulation of response to external stimulus (GO:0032103), phagocytosis (GO:0006909), regulation of angiogenesis (GO:0045765), ECM organization (GO:0030198), regulation of inflammatory response (GO:0050727), response to hypoxia (GO:0001666), cell-matrix adhesion (GO:0007160), and ERK1 and ERK2 cascade (GO:0070371; Supplementary Table S3). The DEmRNAs were also involved in different molecular functions (Figure 3B), primarily in receptor regulator activity (GO:0030545), carbohydrate binding (GO:0030246), glycosaminoglycan binding (GO:0005539), integrin binding (GO:0005178), cytokine activity (GO:0005125), and ECM structural constituent (GO:0005201; Supplementary Table S4). The enrichment analysis identified ECM (GO:0031012), neuron projection membrane (GO:0032589), integrin complex (GO:0008305), protein complex involved in cell adhesion (GO:0098636), and leading edge membrane (GO:0031256) as the cellular components regulated by the DEmRNAs (Figure 3C; Supplementary Table S5).

The KEGG analysis indicated that DEmRNAs were enriched in cytokine‒cytokine receptor interaction (rno04060), phagosome (rno04145), viral protein interaction with cytokine and cytokine receptor (rno04061), ECM‒receptor interaction (rno04512), focal adhesion (rno04510), complement and coagulation cascades (rno04610), the IL-17 signaling pathway (rno04657), the PI3K‒Akt signaling pathway (rno04151), and protein digestion and absorption (rno04974; Figure 3D, Supplementary Table S6).

**FIGURE 4:**
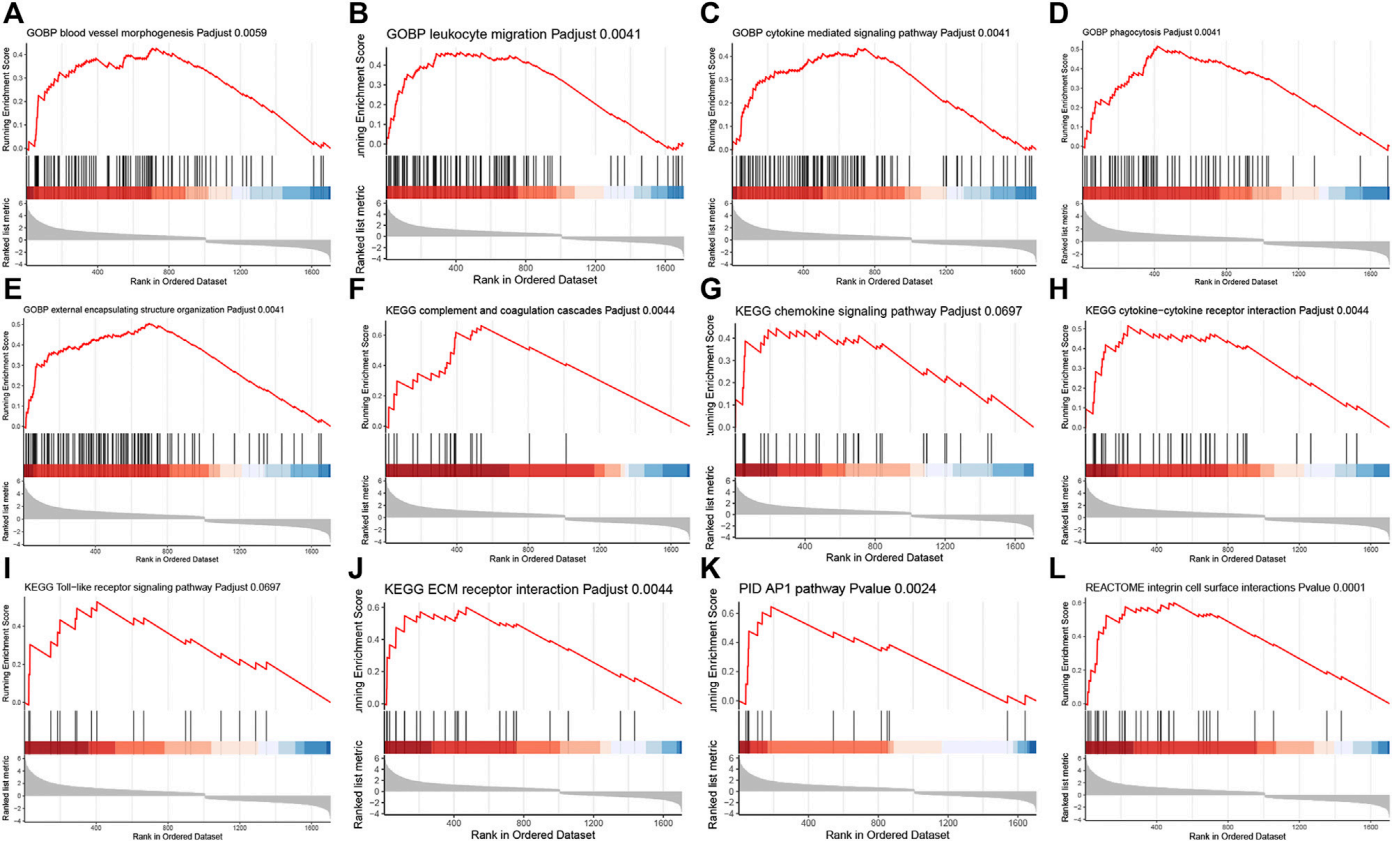
GSEA analysis revealed related biological processes and signaling pathways enriched in the FBR induced by silicone implants. **(A)** Blood vessel morphogenesis. **(B)** Leukocyte migration. **(C)** Cytokine mediated signaling pathway. **(D)** Phagocytosis. **(E)** External encapsulating structure organization. **(F)** Complement and coagulation cascades. **(G)** Chemokine signaling pathway. **(H)** Cytokine-cytokine receptor interaction. **(I)** Toll-like receptor signaling pathway. **(J)** ECM receptor interaction. **(K)** AP1 pathway. **(L)** Integrin cell surface interactions.

In addition, the GSEA was performed to significantly identify the gene sets that were associated with silicone implantation-induced FBR. The results showed that molecular changes during FBR induced by silicone implantation were related to blood vessel morphogenesis, leukocyte migration, cytokine-mediated signaling pathways, phagocytosis, external encapsulating structure organization, complement and coagulation cascades, chemokine signaling pathway, cytokine‒cytokine receptor interaction, the Toll-like receptor signaling pathway, ECM receptor interaction, the AP1 pathway, and integrin cell surface interactions (Figure 4, Supplementary Table S7). Furthermore, the enriched gene sets were similar to those identified in the GO and pathway analysis.

### PPI Network Analysis

**FIGURE 5:**
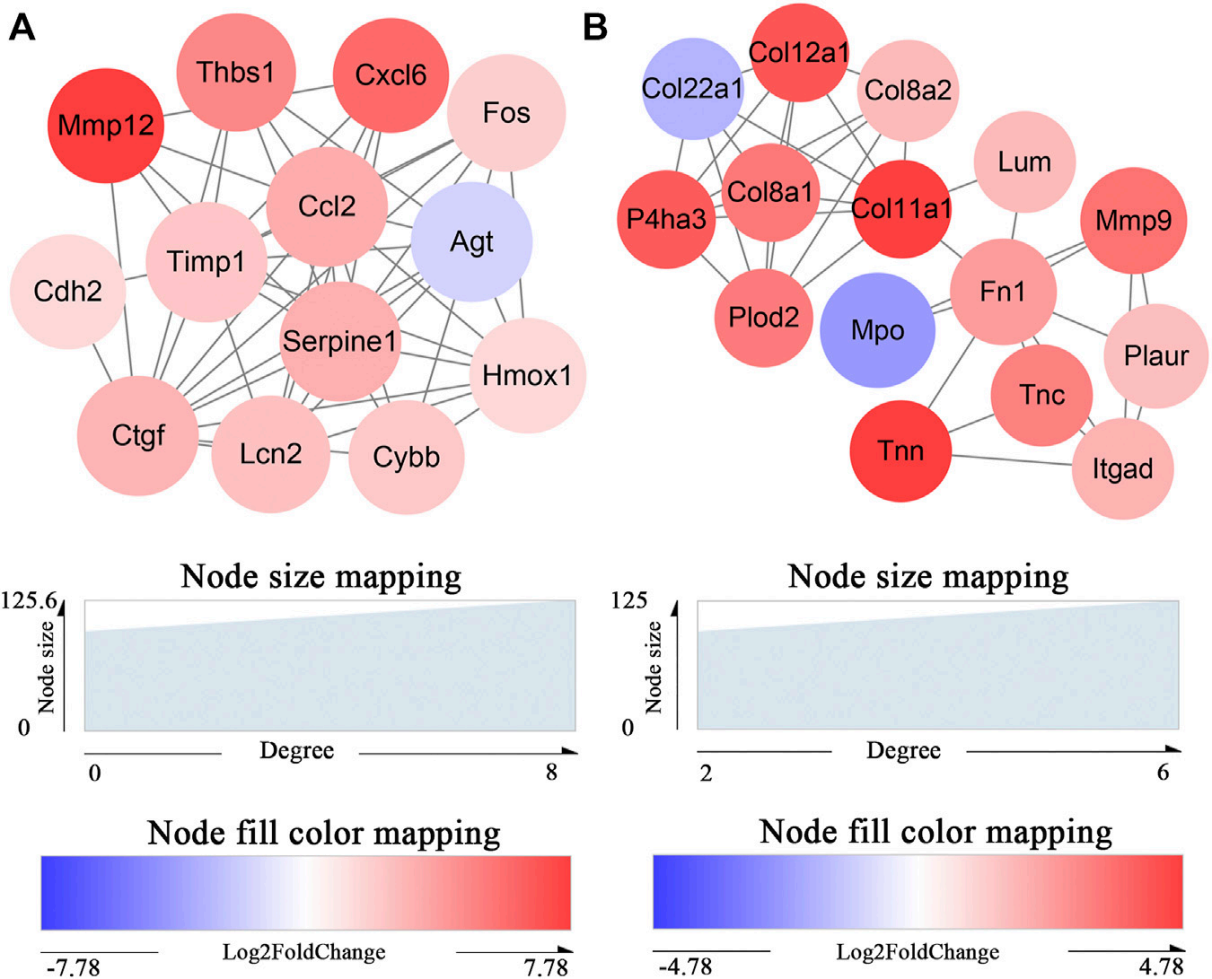
Identification of key protein clusters using string database and MCODE plugin (nodes>10, score>5). **(A)** Color-coded network of protein module 1 and their connection. **(B)** Color-coded network of protein module 2 and their connection. The nodes in **(A) (B)** indicate proteins. The edges represent protein interaction.

The 395 DEmRNAs were imported into the String database, and a PPI network comprising 270 nodes and 766 edges was constructed. Thereafter, we visualized the PPI network in Cytoscape and applied MCODE for further module analysis. We identified two significant gene modules that had a score >5 (Figures 5A,B). Enrichment analysis via ClueGO indicated that gene module 1 primarily played a role in the positive regulation of collagen biosynthetic processes. However, we did not observe any significant GO term associated with the gene module 2 (Supplementary Table S8).

**FIGURE 6:**
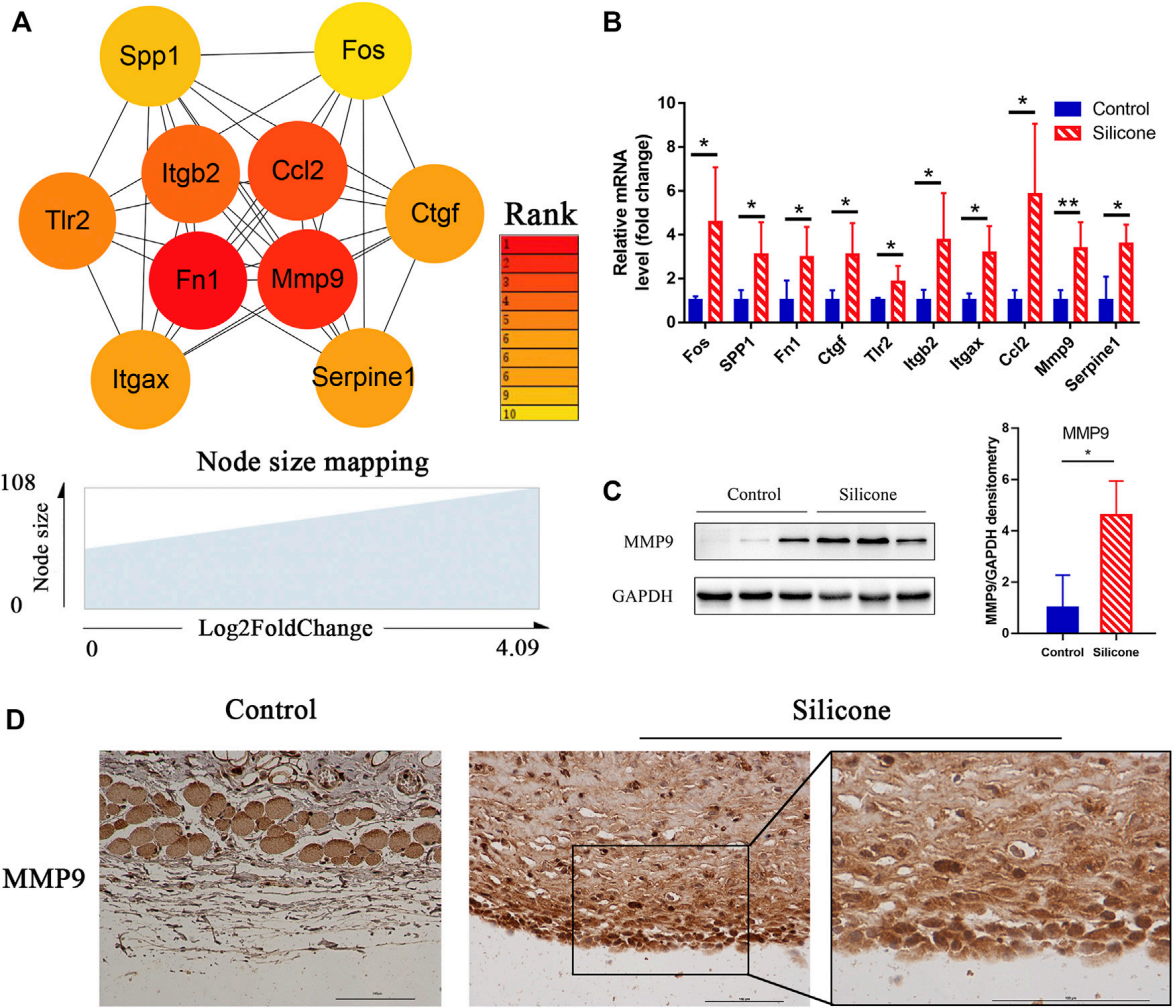
Identification of hub genes related to the silicone-based FBR and their expression analysis. **(A)** Hub genes identified from PPI analysis. **(B)** The relative mRNA levels of FOS, SPP1, FN1, CTGF, TLR2, ITGB2, ITGAX, CCL2, MMP9, and SERPINE1 using qPCR. **(C)** Western blot showed a higher level of MMP9 in silicone group than control group. **(D)** Immunohistochemistry staining showed that MMP9 was mainly located in macrophages and myofibroblasts. Scale bar = 100 μm. *p < 0.05, **p < 0.01.

After analyzing the gene modules, cytohubba was used to identify the hub genes related to silicone implantation: *Fos, Spp1, Fn1, Ctgf, Tlr2, Itgb2, Itgax, Ccl2, Mmp9*, and *Serpine1*. These genes were then ranked by their degree of centrality (Figure 6A). They were primarily involved in several important biological processes, including regulation of collagen biosynthetic processes (GO:0032965), angiogenesis (GO:0001525), the integrin-mediated signaling pathway (GO:0007229), cell-substrate adhesion (GO:0031589), and the Toll-like receptor signaling pathway (rno04620). Furthermore, qRT-PCR revealed a similar trend with the RNA-seq results, which indicated the reliability of the sequencing data (Figure 6B). Interestingly, the four hub genes *Fos, Ctgf, Ccl2*, and *Serpine1* were enriched in gene module 1. In contrast, gene module 2 included the two hub genes *Fn1* and *Mmp9*. Notably, Mmp9 showed a significant elevation in the relative mRNA expression level, further validations were applied to identify the protein expression and location by Western blot and immunohistochemistry. A high MMP9 expression was found in silicone group. And MMP9 is mainly located in macrophages and myofibroblasts, especially those cells close to the silicone implant (Figures 6C,D).

### Construction of the Regulatory TF‒mRNA Interaction Network

**FIGURE 7:**
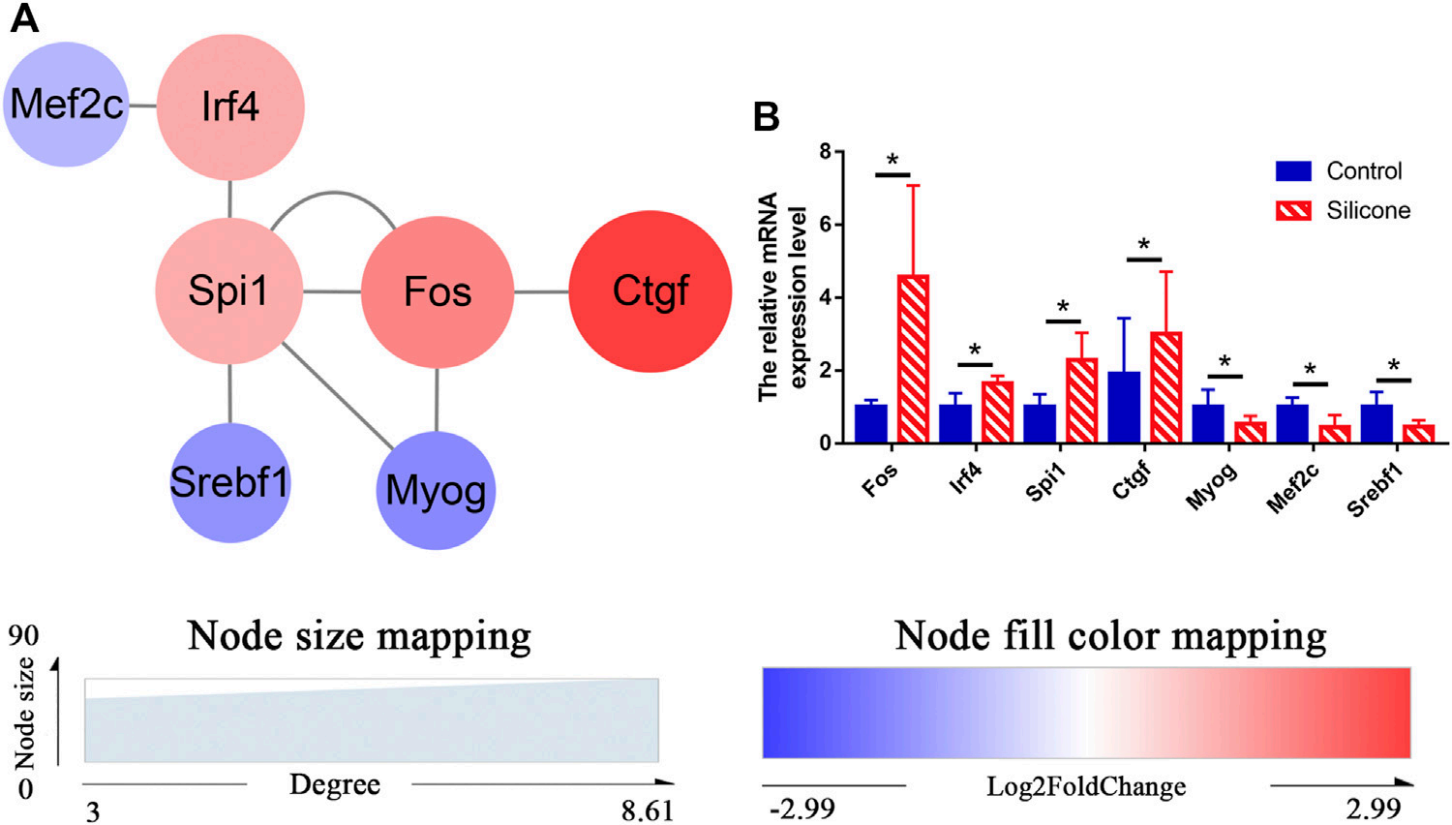
Regulation of TFs-DEmRNAs interactions using transcription factor databases. **(A)** Regulatory networks of TFs-DEmRNAs interactions activated by the FBR to silicone implants. **(B)** Relative levels of mRNA for Fos, Spi1, Irf4, Ctgf, Myog, Mef2c, and Srebf1 using qPCR. *p < 0.05.

To understand complex biological processes, such as FBR and fibrous capsule formation, it is crucial to elucidate and understand their regulatory machinery. We identified 3 TFs (FOS, IRF4, SPI1) and analyzed their target genes by TF database prediction. Overlapping mRNAs between TF target genes and the DEmRNAs were used to reconstruct TF‒mRNA interactions (Figure 7A). We measured the expression levels of 3 TF genes (*Fos, Spi1*, and *Irf4*) and four DEmRNAs (*Myog*, *Ctgf*, *Mef2c*, and *Srebf1*) by qRT-PCR and found a similar mRNA expression pattern as that obtained by RNA-seq (Figure 7B, Supplementary Table S9).

### Molecular Signatures of Important Biological Processes During the FBR

**FIGURE 8:**
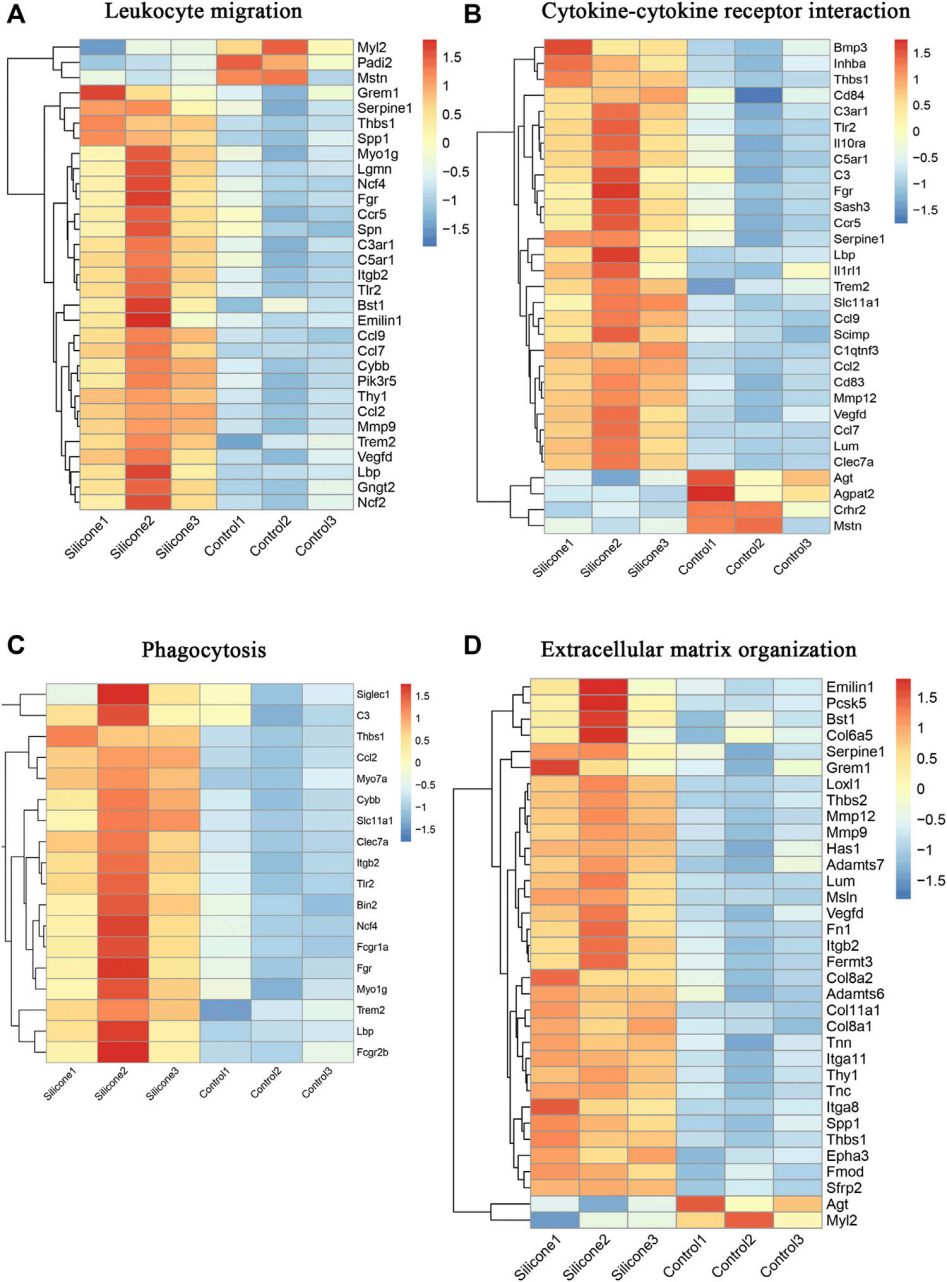
Molecular signatures in several important biological processes in the FBR induced by silicone implantation. **(A)** HeatMap of molecules involved in leukocyte migration. **(B)** HeatMap of molecules involved in cytokine-cytokine receptor interaction. **(C)** HeatMap of molecules involved in phagocytosis. **(D)** HeatMap of molecules involved in extracellular matrix organization.

The GO annotation of the FBR-related transcriptome identified leukocyte migration, cytokine‒cytokine receptor interaction, phagocytosis, and ECM organization as the enriched GO terms. Most of the genes contained in these GO terms were upregulated during the FBR evoked by silicone implants. Furthermore, we also identified the involvement of the hub genes enriched in several important biological processes: *Tlr2, Itgb2, Mmp9, Serpine1*, and *Ccl2* in leukocyte migration (Figure 8A, Supplementary Table S10); *Tlr2, Serpine1*, and *Ccl2* in cytokine‒cytokine receptor interaction (Figure 8B, Supplementary Table S11); *Tlr2, Itgb2*, and *Ccl2* in phagocytosis (Figure 8C, Supplementary Table S12); and *Fn1, Itgb2, Serpine1, Spp1*, and *Mmp9* in ECM organization (Figure 8D, Supplementary Table S13).

## Discussion

Silicone implants can cause adverse reactions in the host tissues. Indeed, fibrous capsule form around the implants, impairing their function, limiting their potential use, and even causing autoimmune diseases (Cohen Tervaert and Kappel, 2013; Colaris et al., 2017). Although many studies have sought to reveal the mechanisms underlying these silicone-related complications (Huang et al., 2017; Klopfleisch and Jung, 2017), little is known about changes in the transcriptome profiles during an FBR induced by silicone implants. Thus, we used a rat model having a subcutaneous silicone implant in the scalp and observed fibrous capsule formation at 30-days post-silicone implantation. Importantly, numerous fibroblasts, myofibroblasts, macrophages, FBGCs, and blood vessels were observed in the capsules. These results confirmed the presence of silicone implant-induced FBR. Based on our high-throughput sequencing data, we comprehensively depicted the transcriptional profiles of the FBR and identified ten hub genes (*Fos, Spp1, Fn1, Ctgf, Tlr2, Itgb2, Itgax, Ccl2, Mmp9*, and *Serpine1*). These genes possibly serve as key molecules in fibrous capsule formation during FBR. Furthermore, we constructed a TF‒mRNA interaction regulatory network. Using cluster analysis, we identified multiple differentially expressed genes involved in several important biological processes, including leukocyte migration, cytokine‒cytokine receptor interaction, phagocytosis, ECM organization, and angiogenesis. KEGG analysis of DEmRNAs revealed potentially significant signaling pathways mainly related to cytokine‒cytokine receptor interaction, chemokine signaling pathway, ECM‒receptor interaction, complement, and coagulation cascades. Hence, our data will contribute to a better understanding of the process of fibrous capsule formation associated with silicone-induced FBR and to the development of effective approaches to mitigate complications induced by the FBR.

Notably, FOS was identified as both a TF and a hub gene; thus, it appears to play a key role in the FBR induced by silicone implantation. It can dimerize with proteins of the JUN family and participate in the formation of the AP-1 transcription factor complex. Once the innate immune system is activated upon silicone implantation, Toll-like receptor signaling is relayed to adapter molecules that eventually activate AP-1, leading to cytokine and chemokine production (Kawai et al., 2004). In addition, it is well known that FOS/AP-1 pathway induces the formation of fibronectin and TGF-β and the deposition of collagen (Palomer et al., 2020). Hence, our results suggest that FOS plays an essential role in controlling inflammation and fibrosis during the silicone-induced FBR.

The glycoprotein SPP1 (secreted phosphoprotein 1) is produced by fibroblasts and activated macrophages in healing wounds, as well as in dendritic, lymphoid, and mononuclear cells of the immune system (Dai et al., 2009). Many studies have shown that SPP1 is highly expressed during the acute phases of inflammation and fibrosis (Morse et al., 2019). Our data further indicated that increased SPP1 expression in an inflammatory response and fibrosis was associated with silicone implant-induced FBR. Furthermore, SPP1 is a key mediator of inflammatory responses and functions as a chemoattractant for immune cells. Indeed, the main role of SPP1 during inflammation is to trigger different leucocytes, induce cytokine secretion, increase macrophage infiltration, induce transforming growth factor (TGF)-β secretion, and promote fibroblast proliferation (Icer and Gezmen-Karadag, 2018). It also upregulates collagen expression in fibroblasts by enhancing TGF-β signaling during fibrosis (Kramerova et al., 2019). In addition, secreted SPP1 promotes angiogenesis by binding to integrin αvβ3 and activating the PI3K‒AKT and ERK pathways in endothelial cells, thereby stimulating VEGF production (Dai et al., 2009). Fibronectins participate in the formation of a collagen-based capsule around the implanted silicone. Fibronectin 1 (FN1) is involved in cell adhesion and migration by promoting leukocytes to migrate through the ECM to the site of inflammation. Connective tissue growth factor (CTGF) is a major connective tissue mitoattractant and is secreted by vascular endothelial cells. Its primary functions are mediating cell adhesion, promoting immune cell recruitment, cytokine production, and angiogenesis. In addition, CTGF is a central mediator of tissue remodeling and fibrosis. It interacts with TGF-β and integrin αvβ3 to accumulate in the ECM and promote fibrosis (Lipson et al., 2012; Henderson and Sheppard, 2013). These findings suggested that CTGF exerts pro-inflammatory effects and stimulates the fibrotic tissue formation around the implant during an FBR.

The activation of Toll-like receptors is important for the activation of innate immunity. Our results showed that Toll-like receptor 2 (*Tlr2*) is one of the hub genes that is upregulated during the FBR. It is a member of the Toll-like receptor family that causes NF-kappa-B activation, cytokine secretion, and an inflammatory response via MYD88 and TRAF6 (Oliveira-Nascimento et al., 2012). Previous studies have demonstrated that *Tlr2* is a hub gene that is likely involved in silicone-induced immune response (Huang et al., 2017). Furthermore, *Tlr2* activation in immune cells during chronic inflammation can contribute to the pathogenesis of chronic diseases (Mahfoud and Petrova Tatiana, 2021), such as autoimmune diseases. In addition, a study has recently revealed that endothelial TLR2 promotes angiogenesis in cancer (Mccoy et al., 2021). The hub gene *Itgb2* encodes a protein that combines with ITGAX to form different heterodimers. The integrins ITGAX/ITGB2 are receptors of fibrinogen and the iC3b fragment of the third complement component. They participate in cell adhesion and phagocytosis during the FBR, and their related downstream pathways involve integrin‒cell surface interactions and ERK signaling.

Matrix metalloproteinases (MMPs) can degrade structural components within the ECM and at the cellular surface. Hence, MMPs cause changes in cellular behavior, such as cell adhesion and migration, and in subsequent pathological responses, such as the FBR. We identified MMP9 as a key modulator of several processes in the FBR and demonstrated that it is activated during capsule formation after silicone implantation. We also identified numerous FBGCs. Consistent with our findings, a previous study has shown that MMP9 is required for the macrophage fusion process and for FBGC formation. Importantly, Mmp9-null mice display abnormalities in collagenous encapsulation, ECM deposition, and blood vessel formation during an FBR (Maclauchlan et al., 2009).

CCL2 has multiple roles in macrophage function. Our data confirmed the upregulation of CCL2 during the FBR. A previous study has shown reduced macrophage fusion, but normal macrophage recruitment, in biomaterials that are subcutaneously implanted in Ccl2-null mice (Kyriakides et al., 2004). These results suggest that MMP9 and CCL2 participate in FBGC formation through macrophage fusion and capsule formation during the silicone-induced FBR.

SERPINE1 is an inhibitor of fibrinolysis that is responsible for skin fibrosis. In fibroblasts, SERPINE1 stimulates collagen accumulation via the SMAD-dependent TGF-β signaling pathway (Ghosh and Vaughan, 2012). Together, these results suggest a possible role of SERPINE1 in the physiological mechanism of capsule formation induced by the FBR to silicone.

Our PPI network analysis revealed several TF‒mRNA interactions that might contribute to the FBR. Of note, IRF4, MEF2C, and SPI1 have been reported to be crucial in the development of autoimmunity (Xu et al., 2012; Li et al., 2020a; Felton et al., 2021). For instance, IRF4 plays essential roles in the activation and differentiation of multiple subsets of B and T cells (Li et al., 2020a; Cook et al., 2020; Felton et al., 2021). SPI1 is a master TF in the differentiation of immune cells and can bind to lineage-defining partners like IRF4/8. (Rothenberg et al., 2019; Li et al., 2020b). Since these cells are crucial to the pathogenesis of autoimmune diseases, IRF4 possibly facilitates the initiation and progression of autoimmune diseases.

MYOG induces fibroblasts to differentiate into myofibroblasts, which are critical for the actuate formation of fibrotic tissue and shrinking of capsules around implants. Interestingly, we found numerous myofibroblasts in the capsules, indicating the participation of MYOG in the silicone-induced capsule formation. Moreover, MYOG can cooperate with MEF2, activating numerous downstream genes to initiate muscle cell differentiation (Lv et al., 2020). Our data shows the decreased expression of MYOG and MEF2C. These results suggest that there may be additional mechanisms regulating myofibroblast differentiation. Therefore, further work should be undertaken to investigate the functions and regulatory networks of MYOG and MEF2C during FBR. However, little information about SREBF1 is currently available to derive preliminary understanding of its function and role in inflammation, fibrosis, and immune-regulation. It is necessary to identify specific cell lines and animals to demonstrate the exact function of SREBF1 when host tissue interacts with silicone implants.

There were some limitations to our study. The mRNA expression levels of the ten hub genes and 3 TFs were verified in the skin tissue of sub-scalp silicone-implanted rat models; however, their expression and function during silicone implant-induced FBR have not been investigated in humans. Nevertheless, our study provides new understanding of TFs and the molecular mechanisms that coordinate the gene expression patterns during an FBR induced by silicone implantation. Moreover, further research should be performed to investigate the effects of these ten hub genes and 3 TFs on the FBR using gene knock-out animals.

## Conclusion

In summary, by using high-throughput technology and comprehensive analysis, we identified ten hub genes (*Fos, Spp1, Fn1, Ctgf, Tlr2, Itgb2, Itgax, Ccl2, Mmp9*, and *Serpine1*) and 3 TFs (FOS, IRF4, SPI1) that are related to the pathophysiology of an FBR induced by silicone implantation. Several biological processes were involved in this silicone-induced FBR, including leukocyte migration, cytokine‒cytokine receptor interaction, angiogenesis, phagocytosis, ECM organization, and regulation of the inflammatory response. However, in the future, we plan to validate our findings in a large quantity of clinical specimens. Further *in vitro* experiments may provide a deeper mechanistic insight into the regulatory relationship underlying the silicone-induced FBR. Nonetheless, the function analysis of these key TFs and hub genes may provide novel therapeutic targets to reduce complications related to silicone implantation.

## Data Availability

The datasets generated during the current study are available in the NCBI repository; the names of the repository and accession number can be found below: NCBI SRA Bio Project, accession no: PRJNA753961.
