# Supplementary material for: Deciphering Key Foreign Body Reaction-Related Transcription Factors and Genes Through Transcriptome Analysis
Source: Front Mol Biosci. 2022 Mar 8;9:843391. doi: 10.3389/fmolb.2022.843391 (PMC8958039; doi:10.3389/fmolb.2022.843391)
Supplement: Supplementary file 1 [file DataSheet2.docx]

Supplementary Material

# Supplementary Materials and Methods

## Ethics Statement

The experimental protocol was approved by the animal experiment ethics committee of the Fourth Military Medical University (Xi’an, China), and animal experiments were performed according to the guidelines of the Animal Care Committee (permit no. IACUC-20120117).

## Animal experiments

Six-week-old male Sprague–Dawley rats were purchased from the Experimental Animal Center of the Fourth Military Medical University and were fed under specific pathogen-free conditions. Briefly, the rats were randomly assigned to two groups, the silicone-implanted group (n = 4) and the control group (n = 4).

A round silicone sheet of 1.0 cm diameter was customized by Wanhe Plastic Materials Co, Ltd (Guangzhou, China). Eight rats were randomly divided into two groups, the silicone-implanted group (n = 4) and the control group (n = 4).

Rats were anesthetized with 2% sodium pentobarbital. The hair in the surgical area was shaved, and the eyes were lubricated with an ophthalmic ointment before surgery. The rats were disinfected with iodophor while in a prone position. A 1.5-cm transverse incision was created at the appropriate cervical dorsal area and a subcutaneous pocket was created under the scalp. The silicone sheet was surgically implanted under the scalp of rats that belonged to the silicone-implanted group, whereas the control group rats did not receive any implants. The day on which the surgery was performed was defined as day 0.

## Tissue collection

At 30-days post-surgery, all rats were anesthetized, and the scalp with the fibrous capsule was dissected out. One part of the scalp tissue sample was fixed in 10% neutral buffered formalin and was embedded in paraffin. From this, 3-μm-thick sections were sliced and prepared for hematoxylin and eosin (H&E)-staining and immunofluorescence. The remaining tissues were stored in RNAlater (Invitrogen, Waltham, MA, USA) at -20°C.

## H&E-staining and immunofluorescence

Tissue sections were stained with H&E according to routine procedures. For immunohistochemistry, tissue sections were submitted to an antigen retrieval step at 96°C for 20 min in citrate buffer at PH 6.0, blocked for 1 h, after which they were incubated with primary antibody overnight at 4°C in a humid chamber. The antibodies were mouse anti-rat CD68 (1:100, ab31630, Abcam, Cambridge, UK), and rabbit anti-rat alpha smooth muscle actin (1:100, 14395-1-AP, α-SMA, ProteinTech Group, Wuhan, China). After washing in PBST (PBS with 0.1% Tween‒20), Fluorescently labeled secondary antibodies (antibody to mouse or rabbit) were applied for 1 h at 37℃ in a humid chamber (1:1000, Invitrogen, Carlsbad, CA, United States). After that, tissue sections were washed for 5 min with PBST, which was repeated three times. Nuclei were counterstained with 10 μg/ml DAPI (Solarbio, Beijing, China). Then, PBST solution was added again to rinse the tissue section three times. Immunofluorescent staining was examined using Nikon laser scanning confocal microscope (Nikon, Tokyo, Japan).

## RNA Extraction and Quality Examination

RNA extraction and quality examination were performed by Annoroad Gene Technology Co. Ltd. (Beijing, China; http://www.annoroad.com/). Total RNA was extracted using Trizol reagent according to the manufacturer’s recommendations. RNA purity was checked using the KaiaoK5500® Spectrophotometer (Kaiao, Beijing, China). RNA integrity and concentration were assessed using the RNA Nano 6000 Assay Kit of the Bioanalyzer 2100 system (Agilent Technologies, Santa Clara, CA, USA).

## Library construction, examination, clustering and sequencing

A total of 2 μg RNA per sample was used to generate cDNA libraries using NEBNext ® Ultra™ RNA Library Prep Kit for Illumina ® (#E7530L, New England BioLabs Inc., Ipswich, MA, USA) following the manufacturer’s recommendations. Briefly, mRNA was purified from total RNA using poly-T oligo-attached magnetic beads. Fragmentation was carried out using divalent cations under elevated temperature in NEBNext First Strand Synthesis Reaction Buffer (5X). First strand cDNA was synthesized using random hexamer primer and RNase H. Second strand cDNA synthesis was subsequently performed using buffer, dNTPs, DNA polymerase I and RNase H. The library fragments were purified with QiaQuick PCR kits and elution with EB buffer, then terminal repair, A-tailing and adapter added were implemented. The aimed products were retrieved and PCR was performed, then the library was completed. The RNA concentration of the library was measured using Qubit® RNA Assay Kit in Qubit® 3.0 for preliminary quantification and then diluted to 1 ng/uL. Insert size was assessed using the Agilent Bioanalyzer 2100 system (Agilent Technologies), and the qualified insert size was accurately quantified using the Step One Plus™ Real-Time PCR system (valid library concentration > 10 nM). Clustering of the index-coded samples was performed on a cBot cluster generation system using HiSeq PE Cluster Kit v4-cBot-HS (Illumina, San Diego, CA, USA) according to the manufacturer’s instructions. One silicone-implanted sample and one control sample were deleted due to the clear separation between them and other samples in their own groups. After cluster generation, the libraries were sequenced using Illumina HiSeq 4000 (Annoroad Co. Ltd.) and 150 bp paired-end reads were generated.

## Data filtering and alignment

In order to guarantee the data quality which was used to analysis, the useful Perl script (https://github.com/mdshw5/fastqp) was used to filter the original data. Removing the adapter, low-quality of bases and N-bases were processed. The reference genomes and the annotation file were downloaded from ENSEMBL database (<http://www.ensembl.org/index.html>). Bowtie2 v2.2.3 was used for building the genome index, and clean data was then aligned to the reference genomes and annotation file (Ensembl, v. Rnor 6.0.87) using HISAT2 v2.1.0. Reads count for each gene in each sample was counted by HTSeq v0.6.0, and FPKM (Fragments Per Kilobase Millon Mapped Reads) was then calculated to estimate the expression level of genes. FPKM could eliminate the effect of sequencing depth and gene length on gene expression levels in each sample.

## Differential gene expression analysis

DEGseq v1.18.0 was used for differential gene expression analysis. DESeq2 estimated the expression level of each gene in each sample by linear regression. The p-value was then calculated via Wald test. Finally, the p-value was corrected by the Benjamini–Hochberg method. Genes with |log2FC| ≥ 1.5 and p < 0.05, q < 0.05 were classified as DEmRNAs. Volcano plots of DEmRNAs were prepared using the “ggplot2” library of the R software. The correlation coefficient of every two samples was calculated using the “pearson” function in the “stats” package, and the results were visualized in R using the “pheatmap” package.

## Principal component analysis (PCA)

PCA is used to reduce the dimensionality of large datasets and increase interpretability while minimizing information loss. PCA of the transcriptome data was performed using the “prcomp” function in “stats” package to assess resemblance between samples. The obtained results were visualized in R using the “scatterplot” package.

## GO and KEGG analysis

Metascape (http://metascape.org)(Zhou et al., 2019) was used to conduct GO and pathway enrichment analysis of DEmRNAs (p-value < 0.01, minimum count of 3, and enrichment factor >1.5). The GO annotations (version:1.2, http://purl.obolibrary.org/obo/go/go-basic.obo) for the biological process, cellular component, and molecular function categories and KEGG pathways (version: 99.0) were enriched based on the Metascape online tool. Functional enrichment analysis was also performed by using GO (version:1.2)/KEGG (version:99.0) tools in Hiplot (https://hiplot.com.cn/advance/clusterprofiler-go-kegg), a comprehensive web platform for scientific data visualization. The most statistically significant term within a cluster was considered to represent the cluster.

## Gene set enrichment analysis (GSEA)

GSEA (https://software.broadinstitute.org/gsea/index.jsp) was performed using GSEA software version 2.2.2.0, which detects whether a series of a priori defined biological processes were enriched in the gene rank derived from DEmRNAs between the silicone group and the control group. We used the H, C2, and C5 collections from the Molecular Signatures Database (MSigDB v5.0). A threshold of p < 0.05 was applied for the analysis.

## PPI Network Analysis

PPI network analysis was performed using the Search Tool for the Retrieval of Interacting Genes (STRING) database (https://string-db.org/). PPIs derived from experiments and databases with a combined score > 0.4 were reserved for further analysis. Cytoscape software version 3.8.0 (<http://www.cytoscape.org/>) was used to visualize PPI networks. To infer more biologically interpretable results, the Molecular Complex Detection (MCODE) tool (https://mcodeinitiative.org/) was used to search for high modularity clusters within the network (degree cutoff = 2, node score cutoff = 0.2, k-score = 2, maximum depth = 100). Clusters (nodes>=10, score>5) were considered significant. CytoHubba (https://apps.cytoscape.org/apps/cytohubba) was utilized to identify the top hub genes. All nodes not connected to the main network were excluded from this analysis to obtain reliable results. To identify pathways associated with DEmRNAs in each cluster, we used the ClueGO (https://apps.cytoscape.org/apps/cluego) plugin of Cytoscape.

## Transcription Factor Identification and TF–mRNA Regulation Relationship Construction

The Animal Transcription Factors Database (TFDB) provides comprehensive annotation and classification of TFs and cofactors; as such, TFDB was employed to identify interactive transcription factors (Hu et al., 2019). Then, ChIP Enrichment Analysis (ChEA): Transcription Factor Binding Site Profiles, JASPAR Predicted Transcription Factor Targets, ENCODE Transcription Factor Targets, and TRANSFAC Curated Transcription Factor Targets were used to predict target genes. The predicted target genes were compared with DEmRNAs to obtain the overlapping DEmRNAs, and the TF–mRNA interactions were visualized by Cytoscape.

## Quantitative reverse transcription polymerase chain reaction (qRT-PCR) analysis

Total RNA from the tissues was extracted with the TRIzol reagent (Invitrogen, Camarillo, CA, USA). Subsequently, 1000 ng of the extracted RNA was reverse transcribed into cDNA using the PrimeScript RT reagent Kit with gDNA Remover (Takara, Shiga, Japan). We performed qRT-PCR using the TB Green Premix Ex Taq II (Takara, Shiga, Japan) on a BIO-RAD CFX Connect Real-Time System (Bio-Rad, Munich, Germany). The relative expression levels of target genes were normalized with that of *GAPDH* using the 2^-ΔΔCt^ method. The hub genes (*Fos, Spp1, Fn1, Ctgf, Tlr2, Itgb2, Itgax, Ccl2,* *Mmp9,* and *Serpine1*) and TFs (FOS, SPI1, IRF4, MYOG, CTGF, MEF2C, and SREBF1) were analyzed by qRT-PCR. The primer sequences are listed in Supplementary Table S1.

## Statistical analysis

The differences in expression between the silicone and control group tissues were calculated using t-test or the Mann–Whitney U test. The results shown in column graphs are presented as the mean ± standard deviation (SD) from at least three experimental repeats. P-values of less than 0.05 were considered statistically significant. The data were plotted using GraphPad Prism 7.0 (GraphPad Software, San Diego, CA, USA).

References:

Hu, H., Miao, Y.-R., Jia, L.-H., Yu, Q.-Y., Zhang, Q., and Guo, A.-Y. (2019). AnimalTFDB 3.0: a comprehensive resource for annotation and prediction of animal transcription factors. *Nucleic Acids Res.* 47**,** D33-D38.

Zhou, Y., Zhou, B., Pache, L., Chang, M., Khodabakhshi, A.H., Tanaseichuk, O., Benner, C., and Chanda, S.K. (2019). Metascape provides a biologist-oriented resource for the analysis of systems-level datasets. *Nat. Commun.* 10**,** 1523.

# Supplementary Figures


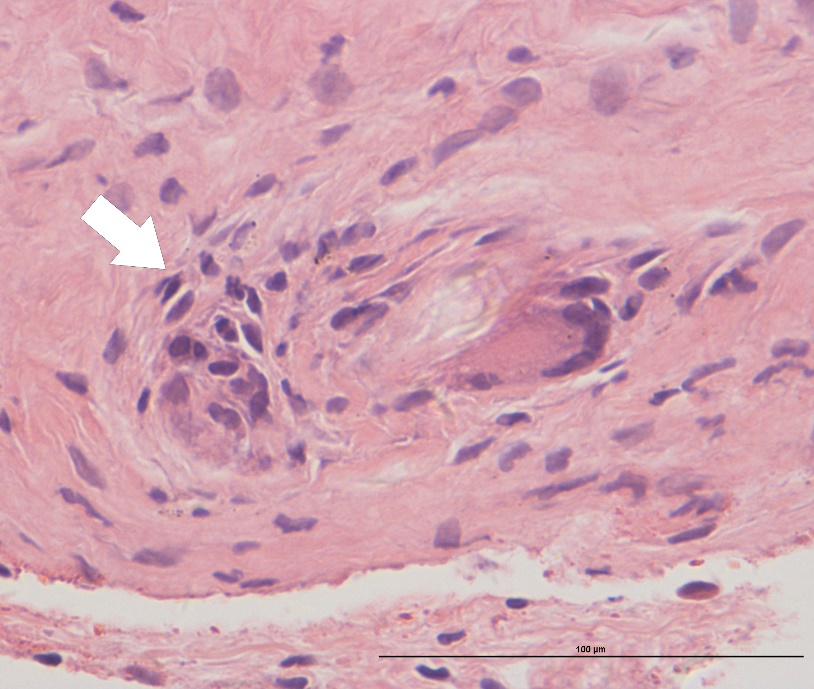


**Supplementary Figure 1.** H&E staining showed foreign body giant cell (FBGC). White arrows point to a FBGC. Scale bar＝100μm.


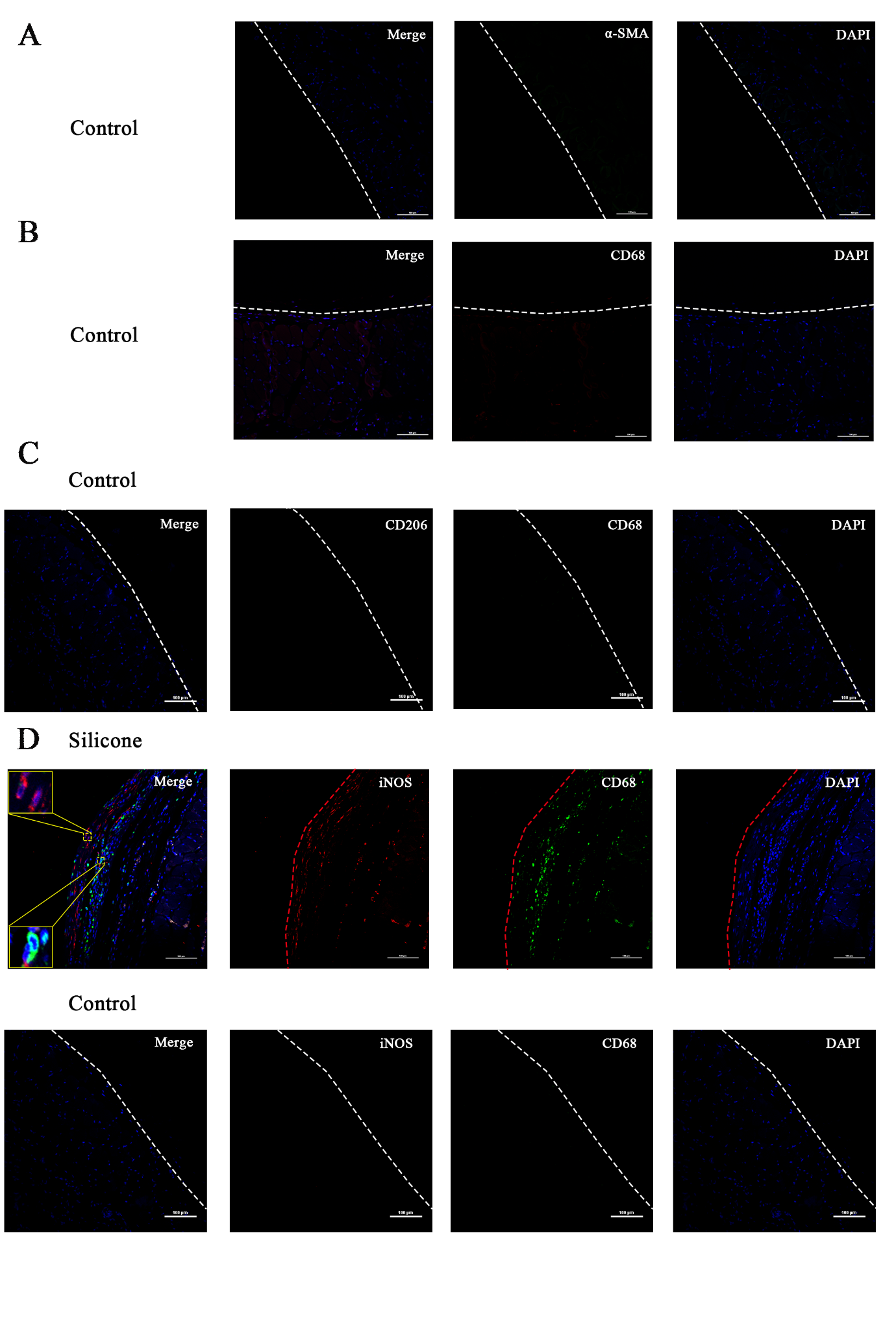


**Supplementary Figure 2.** (A) The immunofluorescence staining of α-SMA in control group. (B) The immunofluorescence staining of CD68 in control group. (C) The immunofluorescence staining of CD68 and CD206 in control group. Because there was no capsule present in the control skin, no α-SMA, CD68, and CD206 were detected. (D) The immunofluorescence staining of CD68 and iNOS in silicone group and control group showed a few CD68^+^/iNOS^+^ double-positive M1 macrophages. Insets show high-magnification images of the CD68^+^(green) or iNOS^+^(red) cells. White dotted line marks the underside of muscle layer. Red dotted line marks the interface between fibrous capsule and silicone implant. Scale bar＝100μm.
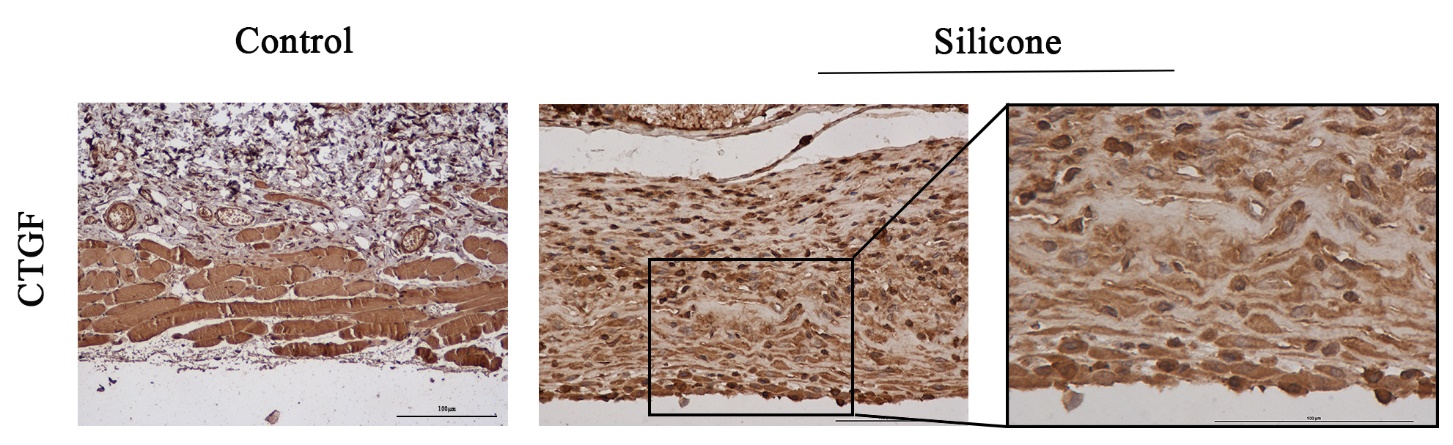


**Supplementary Figure 3.** Immunohistochemistry staining of CTGF showed CTGF markedly increased in fibrous capsule, and mainly localized in the cytoplasm of macrophages and fibroblasts. Scale bar＝100μm.
